# Exploring the Validity of Available Markers and Indices in the Diagnosis of Nonalcoholic Fatty Liver Disease (NAFLD) in People with Type 2 Diabetes in Saudi Arabia

**DOI:** 10.3390/diseases11010010

**Published:** 2023-01-15

**Authors:** Ghada M. A. Ajabnoor, Suhad M. Bahijri, Sumia Mohammad Enani, Lubna Alsheikh, Maimoona Ahmed, Amani Alhozali, Khalid Al-Shali, Basmah Medhat Eldakhakhny, Aliaa A. Alamoudi, Jawaher Al-Ahmadi, Anwar Borai, Alaa Salem Al-Mowallad, Jaakko Tuomilehto

**Affiliations:** 1 Department of Clinical Biochemistry, Faculty of Medicine, King Abdulaziz University, Jeddah 21551, Saudi Arabia; 2 Saudi Diabetes Research Group, Deanship of Scientific Research, King Abdulaziz University, Jeddah 21551, Saudi Arabia; 3 Food, Nutrition and Lifestyle Unit, King Fahd Medical Research Centre, King Abdulaziz University, Jeddah 21551, Saudi Arabia; 4 Department of Food and Nutrition, Faculty of Human Sciences and Design, King Abdulaziz University, Jeddah 21551, Saudi Arabia; 5 Department of Biochemistry, Faculty of Science, King Abdulaziz University, Jeddah 21551, Saudi Arabia; 6 Department of Internal Medicine, Faculty of Medicine, King Abdulaziz University Hospital, King Abdulaziz University, Jeddah 21551, Saudi Arabia; 7 Department of Family Medicine, Faculty of Medicine, King Abdulaziz University, Jeddah 21551, Saudi Arabia; 8 King Abdullah International Medical Research Center, King Saud Bin Abdulaziz University for Health Sciences, King Abdulaziz Medical City, Jeddah 21551, Saudi Arabia; 9 Department of Public Health, University of Helsinki, FI-00014 Helsinki, Finland; 10 Public Health Promotion Unit, Finnish Institute for Health and Welfare, FI-00271 Helsinki, Finland

**Keywords:** nonalcoholic fatty liver disease, type 2 diabetes Saudis, fatty liver index, hepatic steatosis index, NAFLD-liver fat score, triglyceride and glucose index

## Abstract

Nonalcoholic fatty liver disease (NAFLD) is common among Saudi patients with type 2 diabetes (T2DM). However, recommended clinical procedures to detect it are unavailable in many locations. Therefore, better and more available diagnostic biomarkers for NAFLD are needed. Various serum parameters were suggested, and algorithms that employ routine measurements in clinical practice have been developed for the prediction of fat stores in the liver in different populations. However, no such studies have been conducted on Saudis. We aimed to compare selected biochemical markers and calculated indices in T2DM patients diagnosed with NAFLD and patients without NAFLD to find the best markers associated with NAFLD. A cross-sectional study was employed to recruit 67 people with T2DM from endocrine outpatient clinics at King Abdul-Aziz University Hospital. NAFLD was detected by ultrasonography in 28 patients. Demographic information, anthropometric, and blood pressure (BP) measurements were taken. Fasting blood samples were obtained to measure glucose, glycated haemoglobin, lipid profile, liver function tests, and highly sensitive C-reactive protein. Fatty liver index, hepatic steatosis index, NAFLD-liver fat score, and triglyceride and glucose index were calculated. Following stepwise forward likelihood ratio regression with independent variables included in one model using binary logistic regression with age and waist circumference (WC) entered as covariates, elevated diastolic BP and low high-density lipoprotein- cholesterol remained significantly associated with NAFLD (*p* = 0.002 and 0.03, respectively). However, none of the investigated indices could be used to diagnose the disease adequately due to low specificity, even after calculating new cut-off values. Investigating novel markers and adjusting existing equations used to calculate indices to improve sensitivity and specificity in our population is needed.

## 1. Introduction

Nonalcoholic fatty liver disease (NAFLD) is an abnormal condition of the hepatocytes in which fat accumulates without any secondary cause, such as excessive drinking of alcohol, pharmacotherapy that could induce steatosis, or other chronic liver diseases [1]. The pathophysiology of NAFLD begins with fat accumulation in the liver (steatosis), which could develop into nonalcoholic steatohepatitis (NASH) in some people and may be followed by hepatic fibrosis (progressive scarring), culminating subsequently in hepatic cirrhosis, which may then lead to hepatoma [1,2].

A recent meta-analysis estimated the global prevalence of NAFLD to be 25%, with the rise reflecting similar increases in type 2 diabetes (T2DM) and obesity [3,4]. A large Multiethnic Cohort study of 215,000 people found that NAFLD is the most common cause of chronic liver disease, representing 52% of cases across all ethnic groups [5]. Furthermore, NASH is currently the leading indication for liver transplantation among women and the second leading indication for men in the United States [6].

The most important essential risk factor for NAFLD progression is obesity, which is associated with insulin resistance and metabolic syndrome [1,7]. Several studies have found that NALFD is associated with T2DM [7,8,9], and a community-based study of people with T2DM revealed that those with NAFLD had a 2.2-fold increased mortality compared with those without NAFLD [10]. Other studies reported that the presence of NAFLD in people with T2DM may also be linked to an increased risk of cardiovascular disease (CVD) independently of the components of the metabolic syndrome [11,12]. Therefore, the diagnosis and evaluation of fatty liver is an important part of the management of T2DM.

The prevalence of NAFLD was reported to be higher in western countries, approximately 20–30%, compared with 15% in Asian countries [13]. In a recent study published in 2018, the prevalence of NAFLD in Saudi Arabia was found to be 7–10% in the general population [14]. NAFLD, detected by ultrasound, was reported to be common in Saudi patients with T2DM, with most patients presenting with no specific symptoms and the disease being revealed by abnormal liver tests or hepatomegaly [15]. Although hepatic biopsy is considered the gold standard in NASH diagnosis, this invasive procedure may be harmful and not accepted by the patients. Nowadays, liver biopsy is substituted by abdominal ultrasonography or proton magnetic resonance spectroscopy as a non-invasive method for diagnosing NAFLD. However, abdominal ultrasonography, which is more commonly used in Saudi Arabia, cannot diagnose mild steatosis and distinguish between different stages of NAFLD.

Moreover, in some situations, e.g., obesity may interfere with the quality of the images [1,8]. Furthermore, ultrasonography is not widely available in public health care centres, and patients suspected to have NAFLD need to be referred to larger health care centres or hospitals, which increases costs and delays diagnosis, hence the management of the disease. Since the management of T2DM patients diagnosed with NAFLD requires modification of management strategy to avoid further complications, other diagnostic methods, such as serum biomarkers, are preferable and urgently required [16]. Liver enzymes have been suggested as useful markers for diagnosing NAFLD [16,17,18]. In addition, due to the detection of dyslipidemia in Indian patients with NAFLD, serum lipids were suggested as possible disease markers [19]. Various other biomarkers were also suggested, with conflicting results in different populations, including the ratio of AST/ALT, hypoalbuminemia, hyperbilirubinemia, thrombocytopenia, prolonged prothrombin, as well as changes in the level of inflammatory mediators such as an increase in C reactive protein, and tumour necrosis factor-alpha (TNF-α) and decreased adiponectin [20,21]. No such studies have been carried out on the Saudi population, which has already been reported to have a high prevalence of diabetes [22,23]. Moreover, to date, there is no consensus regarding the best biochemical markers which are specific and sensitive for diagnosing NAFLD due to the presence of gaps in the reference interval (RI) database of most of these biomarkers that have been suggested for evaluation of the condition [20,21], especially that differences may be seen in the RIs depending on ethnicity, as well as population’s environmental and dietary conditions [24]. The International Federation for Clinical Chemistry has initiated a global study to establish RI in different populations, and the reference intervals of clinical chemistry analytes for the adult population in Saudi Arabia have been established recently as a part of this study [25].

Various algorithms that employ routine measurements in clinical practice have been developed for the prediction of fat stores in the liver in different populations. Examples of these are the fatty liver index (FLI) [26], hepatic steatosis index (HSI) [27], the NAFLD-liver fat score (NAFLD-LFS) [28], and the serum TG and fasting plasma glucose (FPG) index (TyG) [29]. However, associations between these markers and NAFLD may differ according to ethnicity.

In this pilot study, we aimed to compare easily measured biological and biochemical markers and available indices that use selected, routinely available markers to find indices significantly associated with NAFLD. Based on the strength of previous studies, we choose to include disease duration, HbA1c as a measure of glycaemic control, all liver function tests and lipid profiles which are routinely measured, as well as hs-CRP as a marker of inflammation, and anthropometric measures as markers of obesity. We also measured insulin and fasting plasma glucose to calculate NAFLD-liver fat score (NAFLD-LFS) and fasting plasma glucose (FPG) index. The study was carried out in people with T2DM diagnosed with NAFLD and those without NAFLD; hence, findings can be used to detect Saudi people with T2DM who are most likely to have NAFLD and may be referred to more advanced and expensive testing.

## 2. Materials and Methods

### 2.1. Subjects and Study Design

A cross-sectional study design was employed. The study was approved by The Committee on the Ethics of Human Research at the Faculty of Medicine—King Abdulaziz University, Jeddah, Saudi Arabia, code (No-61-15). People with T2DM were recruited from the outpatient endocrine clinics at King Abdulaziz University Hospital. Diabetic patients with viral hepatitis, hemochromatosis, Wilson’s disease, autoimmune hepatitis, primary biliary cirrhosis, sclerosing cholangitis, biliary obstruction, alpha-1 antitrypsin deficiency, ischemic cardiac or cerebrovascular disease, impaired renal function, malignancies, or alcohol consumption were excluded from our study. Suitable patients who agreed to join the study signed written informed consent. The study lasted from April 2015 to April 2016. Participating patients were interviewed to fill in a predesigned questionnaire which included socio-demographic information, medical history of chronic diseases, as well as drugs used. Blood pressure (BP) was then measured by a standard mercury sphygmomanometer with the cuff on the participant’s right upper arm using standardised techniques based on international recommendations [30]. By following the standard methods and using standardised equipment, anthropometric measurements, height, weight, and hip, waist, and neck circumferences (HC, WC, and NC, respectively) were taken. Body mass index (BMI) was calculated as height in meters divided by weight in kilograms squared. Screening for NAFLD was conducted using a sensitive abdominal ultrasound machine (ACUSON X300™ ultrasound system, premium edition (PE) by Simenes, New York, NY, USA). Fatty liver was diagnosed in the presence of increased liver echogenicity compared to the spleen or the kidneys. Based on the results, participants were then categorised into cases (those with NAFLD) and controls (those without NAFLD).

Fasting blood samples were obtained from all participants. Biochemical analyses were measured in the Clinical Chemistry laboratory at the National Guard Hospital, King Abdul-Aziz Medical City in Jeddah, using Abbott Architect c8000 autoanalyser by Abbott, Illinois, U.S.A (spectrophotometer for serum albumin, alanine transaminase (ALT), aspartate aminotransferase (AST), gamma-glutamyl transferase (GGT), alkaline phosphatase (ALP), total bilirubin, direct bilirubin, triglycerides, high-density lipoproteins cholesterol (HDL-C), total cholesterol, plasma glucose, and immunoturbidimetric determination methods for serum highly sensitive C-reactive protein (hs-CRP). Serum insulin measurement was performed on Abbott Architect i2000 autoanalyser- Abbott, Illinois, U.S.A, by using the method of a chemiluminescent microparticle immunoassay (CMIA). All procedures were performed according to provided instructions, employing specific principles for each analyte. Serum LDL-cholesterol (LDL-C) was calculated using the Friedewald equation [31]. Abnormal values in serum lipids were defined as total cholesterol ≥ 5.18 mmol/L, HDL-C < 1.04 mmol/L for men and <1.3 mmol/L for women, LDL-C ≥ 3.37 mmol/L, triglycerides ≥ 1.7 mmol/L or treatment with lipid-lowering drugs with all lipid levels in the normal range [32,33]. The Saudi reference values were used to define abnormal values for other biochemical variables [25]. The target for acceptable glycemic control was considered as HbA 1c ≤ 7%, or fasting plasma glucose of 4.4–7.2 mmol/L [34]. Serum hsCRP levels were classified as low < 1, intermediate = 1–3, with >3 mg/L as high-risk groups for CVD according to recommendations by the AHA/CDC (American Heart Association/Centres for Disease Control) Working Group on markers of inflammation in CVD [35].

### 2.2. Indices for the Prediction of NAFLD

Fatty liver index (FLI), hepatic steatosis index (HSI), NAFLD-liver fat score (NAFLD-LFS), and fasting plasma glucose (FPG) index (TyG) were measured as follows:

FLI = (e 0.953 × loge (TG) + 0.139 × BMI + 0.718 × loge (ggt) + 0.053 × WC − 15.745)/(1 + e 0.953 × loge (TG) + 0.139 × BMI + 0.718 × loge (ggt) + 0.053 × WC − 15.745) × 100 with a cut-off point of ≥60 [26].

HSI = 8 × (ALT/AST ratio) + BMI (+2, if female; +2, if diabetes mellitus) with a cut-off point of >36 [27].

NAFLD-LFS = −2.89 + 1.18 ⁎ metabolic syndrome (yes = 1/no = 0) + 0.45  ×  type 2 diabetes (yes = 2/no =  0) + 0.15 × fS-insulin (mU/L) + 0.04  × fS-AST (U/L) − 0.94 ×  AST/ALT with a cutoff point of >−0.64 [28].

TyG = Ln [TG (mg/dL) FPG (mg/dL)/2] with a cut-off point of >8.6 [36].

### 2.3. Statistical Analysis

The data obtained were analysed using SPSS version 20. Independent T-test was used to compare the means of the two groups of people with T2DM with and without NAFLD, and when normality was not confirmed, the Mann–Whitney U-test was used. The Chi-square test was used to compare the distribution of categorical variables between the two groups.

Stepwise logistic regression models unadjusted and adjusted for age and BMI were used to identify the variables significantly associated with the NAFLD. Independent factors included WC, clinical and biochemical variables. Related independent variables were included based on the stepwise forward likelihood ratio binary logistic regression [37]. Regression coefficients with their 95% CI and partial correlation coefficients were presented. The association between continuous variables associated with NAFLD was tested with Spearman’s correlation. The receiver operating characteristics (ROC) curve and the area under the curve (AUC) were used to assess different indices’ ability to identify NAFLD. The optimal cut-off values for the identification of NAFLD were determined from the ROC curve. A *p*-value < 0.05 (two-sided test) was accepted as statistically significant.

## 3. Results

### 3.1. Demographic, Anthropometric, and Clinical Characteristics

A total of 67 people with T2DM were included in the study. Following ultrasonography, 28 of them were found to have NAFLD. The demographic, anthropometric, and clinical characteristics of both groups are presented in Table 1.

Mean age, sex distribution, BMI, and duration of NALFD were not significantly different between participants with and without NAFLD. However, means of WC, HC, and WC height were significantly higher in women with NAFLD compared with those without. All people who had NAFLD had high WC (>80 cm for women and >94 cm for men) and >0.5 WC: Ht ratio. In addition, there was a significantly higher percentage of hypertension among those with NAFLD.

### 3.2. Biochemical Characteristics

Serum biochemical variables of diabetic patients with NAFLD and those without were compared and presented in Table 2. The means of blood lipids did not differ significantly between diabetic patients with and without NAFLD. However, low HDL-c was significantly (*p* = 0.001) more prevalent among those with NAFLD (67.9%) than in those without NAFLD (25.6%) (Table 2).

The mean levels of all measured liver enzymes were higher in patients with NAFLD than those without. However, this was statistically significant for AST (*p* = 0.02) and ALP (*p* = 0.03) (Table 2). High AST and high ALT were significantly more prevalent among those with NAFLD. Very high values of GGT were only found in people with NAFLD.

There was no difference in the mean levels of HbA1c% or FPG between diabetic patients with and without NAFLD. However, those with NAFLD had significantly higher serum insulin levels (*p* = 0.004; Table 2).

Even though some biochemical variables had different cut-off values for men and women, the association between all biochemical variables and NAFLD was not influenced by sex.

**Figure 1:**
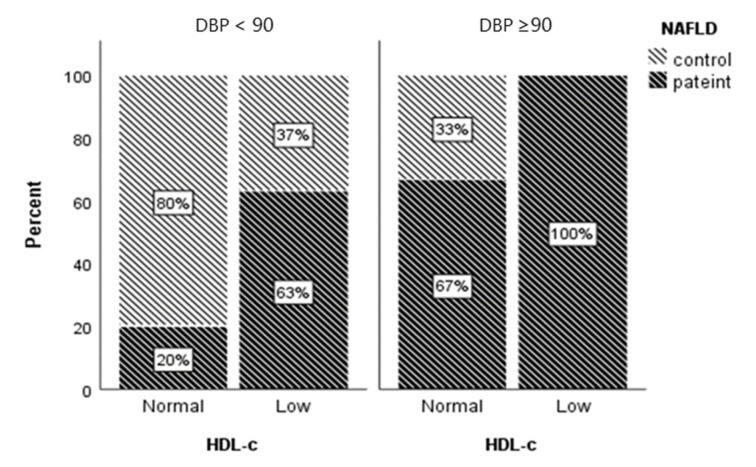
Percentage of NAFLD patients in DBP and HDL groups. Low HDL-c (<1.04 mmol/L for men and <1.3 mmol/L for women).

After adjusting for age and WC as covariates in stepwise forward logistic regression analysis, high DBP and low HDL were found to be the predictors of NAFLD (Table 3); all patients who had both high DBP and low HDL-c had NAFLD (Figure 1).

The calculated means (±SD) of fatty liver indices in T2DM patients with and without NAFLD and the number of patients with high values are presented in Table 4. After applying the cut-off points suggesting NAFLD, all patients who truly had NAFLD were classified as having NAFLD based on FLI and HSI, 96.2% based on NAFLD-LFS, and none based on the TyG index. On the other hand, 87.2% of diabetic patients who truly did not have NAFLD were classified to have NAFLD based on FLI (sensitivity = 1, specificity = 0.13), 89.7% based on HSI (sensitivity = 1, specificity = 0.103), 82.1% based on NAFLD-LFS (sensitivity = 0.962, specificity = 0.154) and none based on TyG index. These proportions were not significantly different between the two groups by NALFD (Table 4). However, patients with NAFLD had a mean FLI of 95.9 ± 6.6, mean HSI of 56.7 ± 9.1, and mean NAFLD-FLS of 2.92 ± 4.3, which were significantly higher than values for patients without NAFLD as these scores were 82.5 ± 24.3, 50.7 ± 9.9 and 0.56 ± 1.75, respectively (*p* < 0.001, *p* < 0.05 and *p* = 0.001, respectively; Table 4).

**Figure 2:**
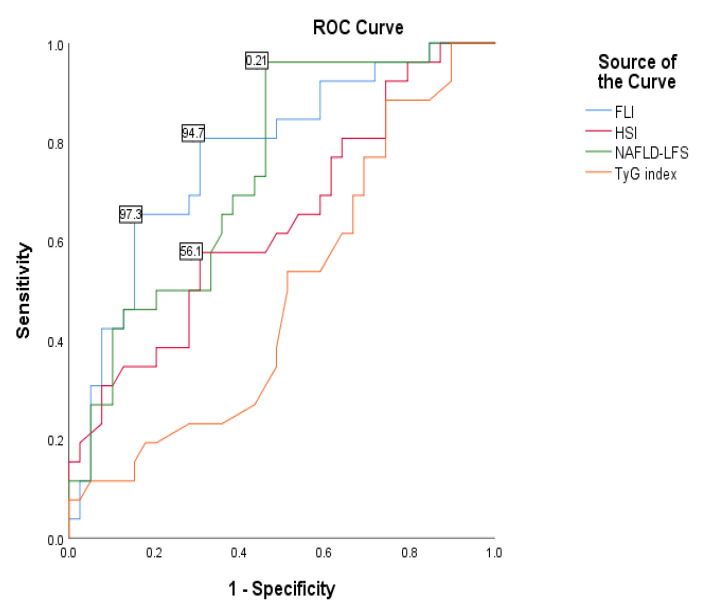
Receiver operating characteristic (ROC) curves for fatty liver index (FLI), hepatic steatosis index (HIS), NAFLD-liver fat score (NAFLD-LFS), and triglyceride and glucose index (TyG) in patients with type 2 diabetes.

The Receiver operating characteristic (ROC) curves for the four studied indices are shown in Figure 2, and the area under these curves (AUC) as well as the 95% CI are presented in Table 5.

Among all the four indices studied, FLI and NAFLD-LFS had the highest AUC to identify NAFLD in diabetic patients [AUCFLI: 0.774 (95% CI: 0.657–0.891), *p* < 0.001; and AUC NAFLD-LFS: 0.74 (95% CI: 0.620, 0.86), *p* = 0.001; Table 5 and Figure 1, both are reasonable but not excellent (>0.8). The ability of HSI to predict NAFLD was not good [AUCHSI: 0.64 (95% CI: 0.501, 0.778), *p* = 0.058; Table 5 and Figure 1.

The optimal cut-off values for liver fat indices and using these calculated cut-off values, their sensitivity and specificity, and positive and negative likelihood ratios (PLR and NLR) for the identification of NAFLD in diabetic patients are presented in Table 6.

The highest sensitivity was found when NAFLD-LFS was used (sensitivity = 0.962). However, its specificity was the lowest compared to the other indices (specificity = 0.538). FLI is more promising, with an acceptable sensitivity of 0.821 and specificity of 0.692. HSI shows the lowest sensitivity, but its specificity is equal to that of FLI (Table 6).

## 4. Discussion

Nonalcoholic fatty liver disease (NAFLD) is the most widespread form of chronic liver disease [3]. Studies on various populations have indicated a rise in the incidence of NAFLD in parallel with the increase in the prevalence of T2DM [38]. Because NAFLD and type 2 diabetes mellitus (T2DM) share the risk factors of excess adiposity and insulin resistance, they appear to coexist together [20]. Indeed, it has been reported that there is a high prevalence of NAFLD among T2DM patients [3,7,9]. Therefore, it was proposed that T2DM forms a route to the development of liver disease, more than double the risk of developing NAFLD and later more advanced liver disease [39]. The global pooled prevalence of NAFLD in T2DM has been reported to be 59.67% [40], while in the city of Abha, south of Saudi Arabia. NAFLD prevalence was estimated to be 72.8% among T2DM patients [41], while it was reported to be 79.3% in a recent study conducted in Riyadh [42]. This high incidence of NAFLD among T2DM patients, as well as the need for better detection methods of this often silent complication of diabetes to optimise management, we aimed, this pilot study In this pilot study, we aimed at comparing routinely available markers as well as available indices based on easily measurable biological and biochemical markers for the prediction of NAFLD in patients with T2DM and investigate which of them is/are significantly associated with NAFLD, and hence can be used to identify people most likely to have the condition, and as the second step to be referred for confirmatory procedures. We found that NAFLD in people with T2DM was associated with a significantly higher DBP and lower HDL-C after adjusting for possible confounding factors such as age and WC. However, age and disease duration were not contributing factors to the development of the disease. The study in Riyadh [42] also reported no difference in mean age between T2DM patients with NAFLD and those without NAFLD. In addition, among all four studied indices, FLI and NAFLD-LFS had the highest AUC to identify NAFLD in diabetic patients, and there was a non-significant trend for the ability of HIS. However, they showed unacceptably low specificity, leading to many false positive cases when used for diagnosing NAFLD.

ALT, AST, ALP, and GGT are known markers of liver dysfunction, are simple to measure, and are suggested as useful markers for the diagnosis of NAFLD [8,19,20]. Both AST and ALT are located in the hepatocytes, so when hepatocytes are injured, as in NAFLD, they will be released into the blood circulation and found elevated in serum [19]. On the other hand, ALP and GGT are usually elevated in obstructive liver disease. When we compared the means of the four liver enzymes for diabetic people with NAFLD and without NAFLD, we found that the mean levels of all four enzymes were higher in diabetic patients with NAFLD. However, this was statistically significant only for AST (*p* = 0.02) and ALP (*p* = 0.03), and the prevalence of high AST and ALT was significantly higher among those with NAFLD. In addition, very high values of GGT were only found in people with NAFLD. However, higher than normal AST, ALP and GGT were found in both groups of people with T2DM patients with or without NAFLD. In another study, elevated serum GGT was the most common laboratory abnormality found in patients with NAFLD, followed by high ALT [21]. In contrast, another study showed a weak association between ALT and the presence of NAFLD [43]. Indeed, several studies [44,45,46] have proposed that GGT may be a simple and reliable marker of hepatic fat deposition and hepatic steatosis, which can lead to hepatic IR, and hence could be considered as a surrogate marker of NAFLD [45,47]. It was also proposed that increased liver fat deposition induces hepatocellular damage, thus simulating the synthesis of GGT in the epithelial cells of the intrahepatic duct [48]. Indeed, various studies reported its value as a risk predictor of vascular injury in metabolic diseases [49,50,51,52]. Age, and excess weight, especially abdominal obesity, are associated with increased liver enzyme levels [17,53]. Therefore, to evaluate the true association between these enzymes with NAFLD, we used adjustment for age and WC; following adjustment all four enzymes, including GGT lost their association with NAFLD. The differences between our results and those of other studies could be due to differences in diagnosing criteria, ethnicity, environmental factors, and statistical analyses of the studied populations [4,8,12,13]. We want to emphasise that no such study has been conducted before on Saudi people, although elevated levels of liver enzymes were commonly believed by Saudi practitioners to indicate the presence of NAFLD.

In an earlier study on T2DM patients, we reported a correlation between GGT level and dyslipidemia [54]. Unfavourable lipid profiles in patients with NAFLD had been reported in previous studies [55]. Dyslipidemia was suggested to be due to the increased production of very low-density lipoprotein particles (VLDL) and dysregulated clearance of lipoproteins from the blood circulation by the liver [56]. In our study, the means of TG, TC, LDL-C, and HDL-C were not significantly different between the T2DM patients with and without NAFLD. In addition, as seen for liver enzymes, an overlap of ranges occurred, and of lipoproteins, only low HDL-C was found to be more common among NAFLD patients, and it remained significant after adjustments. Notably, an earlier German study on people with T2DMreported that fatty liver was found to be significantly associated with low HDL-C, and in particular lower levels of HDL2-C, and that elevated TG was not associated with the disease after adjustment for confounding factors [56].

Using the previously determined cut-off values, FLI, NAFLD-LFS, and HSI showed a high sensitivity to identify NAFLD in Saudi people with T2DM. However, all three indices were non-specific, suggesting a limited diagnostic utility of the FLI, NAFLD-LFS, and HSI in people with T2DM to predict NAFLD. In keeping with our findings, a previous study reported that FLI had limited detection and quantification ability of hepatic steatosis in obese people as it had a low AUC (0.67) with an optimal sensitivity (81%) but low specificity (49%) [57]. The poor specificity of the FLI, NAFLD-LFS, and HSI might be due to the inclusion of variables in these indices that are not directly reflective of hepatic steatosis in diabetic patients. For example, FLI and HSI included BMI and/or ALT and AST as components. However, these variables were not independent predictors of NAFLD in our study. In addition, the difference in the diagnostic ability of these indices is likely to relate to differences in populations and the underlying conditions and prevalence of diabetes. For instance, the FLI was developed as an algorithm to detect NAFLD in the general population using a cohort of Italian individuals with and without liver diseases, whereas, in our Saudi study, only people with T2DM were included. Following the calculation of the best cut-off values, the specificity improved considerably but remained still below an acceptable level. Moreover, as a result, the sensitivity decreased in all three cases, with HSI becoming unacceptably low. All studied indices were developed for other populations, and a modification to the suggested equations might be needed to improve their utility elsewhere.

In this study, the increased risk of NAFLD was not associated with the duration of diabetes, in contrast to an earlier report from India [19]. On the other hand, central obesity was found to be associated with NAFLD, in agreement with findings from previous studies reporting that the most important risk factors for NAFLD progression were central obesity, insulin resistance associated with it, and metabolic syndrome [1,5,9].

Previous studies found that NAFLD increased the release of CRP from the liver to blood circulation [58], and it was proposed that serum highly sensitive CRP (hs-CRP) is a good method to reveal possible NAFLD [59]. Our results showed that the mean hs-CRP serum level was significantly elevated in people with T2DM with NAFLD compared to those without NAFLD, but after multivariable adjustment, hs-CRP lost its significant association with NAFLD. This was not a surprise since hs-CRP is a non-specific marker of several other cardio-metabolic disorders. An independent association of HbA1c with NAFLD was reported in a Chinese population [60]. However, our study in people with T2DM did not find a difference in HbA1c between the two groups by NAFLD status. Hence, it appears that in people with T2DM, HbA1c is not a predictive marker of NAFLD.

## 5. Conclusions

In conclusion, our study showed that age and disease duration were not contributing factors to the development of NAFLD in Saudi people with T2DM. An association was found between NAFLD and some components of the metabolic syndrome, namely increased DBP and decreased HDL-C. Indeed, The European Association for the Study of the Liver 2016 Guidelines actually recommends routine screening for NAFLD with liver enzymes and/or ultrasound in all people with obesity or metabolic syndrome [61]. However, none of the investigated indices can be used to actually diagnose the disease adequately due to low specificity, even after calculating new cut-off values. Investigating new and novel markers and adjusting existing equations used to calculate indices to improve sensitivity and specificity in our population will be carried out in future studies.

The strength of our study lies in being the first in our population to investigate the topic and provide some indications of the characteristics of NAFLD in Saudi people with T2DM in the population where the prevalence of T2DM is high. However, larger studies, which include other markers, are needed for firmer conclusions. Another advantage of our study is that rigorous statistical analysis was performed, thus allowing adjustment for factors affecting levels of measured variables and providing more accurate associations with NAFLD. We hope that these results will help design a bigger study to develop a risk score for NAFLD in Saudis.

The main limitation of this study is its cross-sectional design, which allows the association to be detected, but does not affirm causation or the time course of the noted biochemical abnormalities. Cohort studies are needed to reach the correct sequence of events in disease pathophysiology. Another limitation is the relatively small sample size. However, as a pilot study, the numbers were quite adequate to show associations of some measured variables with NAFLD. Statistical analyses enabled us to reduce the number of investigated analytical variables that may be used in a larger study to develop risk scores for NAFLD among Saudis.

Considering the high prevalence of T2DM in the Kingdom of Saudi Arabia, it might be prudent that, in addition to the routinely recommended measurements of BP, glycemic control and lipid profile, waist circumference should be measured. Then, it might be appropriate to refer patients with low HDL-C, high WC, and DBP to ultrasonography for diagnostic testing of NAFLD. Once a diagnosis of NAFLD is confirmed, more intensive therapeutic interventions are necessary to avoid complications and poor prognosis. Furthermore, the methods used for diagnosing and evaluating NAFLD can also be used to monitor the efficacy of intervention or therapy for NAFLD.

This should help decrease the cost of unnecessary testing and referrals and maintain the integrity of given health care, especially since there is an expected increase in the prevalence of obesity and obesity-related diseases, including T2DM and NAFLD, so that by 2040, the predicted direct health care costs of these diseases are estimated to be 127,956,508,540 [±51,882,446] USD [62].

## Figures and Tables

**Table 1:** Demographic, anthropometric, and clinical characteristics of people with T2DM with and without NAFLD.

	T2DM without NAFLD N = 39	T2DM with NAFLD N = 28	*p* Value
Age (years) (Mean ± SD)	57.0 ± 10.6	57.4 ± 11.9	0.94
**Sex (n,% )**			
Men	16, 41%	6, 21.4%	0.092
Women	23, 59%	22, 78.6%	
**BMI (%)**			
Normal (BMI = 18.5−24.9)	10.3%	0	0.21
Overweight (BMI = 25−29.9)	7.7%	7.1%	
Obese (BMI > 30)	82.1%	92.9%	
**BMI (Mean ± SD)**			
Men	32.3 ± 56.5	37.1 ± 5.7	0.13
Women	43.9 ± 7.0	46.9 ± 8.00	0.18
**Weight (kg) (Mean ± SD)**			
Men	86.2 ± 26.4	97.0 ± 21.9	0.38
Women	72.5 ± 11.4	87.9 ± 14.3	**<0.001**
**WC (cm) (Mean ± SD)**			
Men	107.2 ± 11.9	115.2 ± 16.7	0.22
Women	97.8 ± 13.3	113.4 ± 8.4	**<0.001**
**HC (cm) (Mean ± SD)**			
Men	107.8 ± 9.9	117.0 ± 16.9	0.13
Women	105.5 ± 9.4	118.3 ± 10.7	**<0.001**
**WC:HC (Mean ± SD)**			
Men	0.99 ± 0.04	0.99 ± 0.04	0.67
Women	0.93 ± 0.10	0.97 ± 0.09	0.19
**WC: height (Mean ± SD)**			
Men	0.65 ± 0.06	0.69 ± 0.10	0.23
Women	0.64 ± 0.08	0.72 ± 0.06	**<0.001**
**NC (cm) (Mean ± SD)**			
Men	39.6 ± 3.7	42.5 ± 4.8	0.16
Women	36.1 ± 4.1	38.0 ± 4.1	0.118
**Duration of diagnosed T2DM (%)**			
10 years or under	66.7%	50.0%	0.17
More than 10 years	33.3%	50.0%	
**SBP (%)**			
Equal or more than 140 mm\Hg or on medication	66.7%	85.7%	0.08
**DBP (%)**			
Equal or more than 90 mm\Hg or on medication	51.3%	85.7%	**0.003**
**Hypertension (%)**	51.3%	82.1%	**0.009**

Body mass index (BMI), Waist circumference (WC), Hip circumference (HC), Neck circumference (NC), Type 2 diabetes mellitus (T2DM), Systolic blood pressure (SBP), diastolic blood pressure (DBP). Statistically significant values are shown in bold font.

**Table 2:** Biochemical characteristics of T2DM patients with and without NAFLD.

Biochemical Variables	T2DM without NAFLD N = 39	T2DM with NAFLD N = 28	*p* Value
**Serum total cholesterol (mmol/L)**			
Mean ± SD (Actual range)	4.99 ± 1.32 (2.35–10.17)	4.71 ± 1.35 (2.9–8.25)	0.39 ^a^
Patients with a value ≥5.18 mmol/L or on drug treatment (%)	16 (41.0%)	10 (35.7%)	0.66 ^b^
**Serum HDL-C (mmol/L)**			
Mean ± SD (Actual range)	1.33 ± 0.28 (0.76–2.05)	1.22 ± 0.30 (0.87–2.37)	0.15 ^a^
Patients with a value <1.04 mmol/L for men and <1.3 mmol/L for women or on drug treatment (%)	10 (25.6%)	19 (67.9%)	**0.001** ^ b^
**LDL-C (mmol/L)**			
Mean ± SD (Actual range)	3.31 ± 1.18 (1.17–7.65)	3.05 ± 1.16 (1.45–6.07)	0.38 ^a^
Patients with a value ≥3.37 mmol/L or on drug treatment (%)	16 (41.0%)	10 (35.7%)	0.66 ^b^
**Triglycerides (mmol/L)**			
Mean ± SD (Actual range)	1.79 ± 0.78 (0.54–4.56)	2.17 ± 1.24 (1.02–5.94)	0.37 ^a^
Patients with a value ≥1.7 mmol/L or on drug treatment (%)	17 (43.6%)	15 (53.6%)	0.42 ^b^
**AST (U/L)**			
Mean ± SD (Actual range)	15.82 ± 4.99 (9–32)	21.32 ± 11.2 (7–55)	**0.02** ^ a^
Patients with a value >28 for men and >23 U/L for women (%)	1 (2.6%)	7 (25.0%)	**0.005** ^ b^
**ALT (U/L)**			
Mean ± SD (Actual range)	16.15 ± 7.2 (6–42)	22.75 ± 16.59 (8–71)	0.11 ^a^
Patients with a value >44 for men and >28 U/L for women (%)	0	5 (17.9%)	**0.006** ^ b^
**ALP (U/L)**			
Mean ± SD (Actual range)	68.54 ± 21.8 (31–138)	81.61 ± 27.25 (36–150)	**0.03** ^ a^
Patients with a value >114 U/L (%)	1 (2.6%)	3 (10.7%)	0.17 ^b^
**GGT (U/L)**			
Mean ± SD (Actual range)	32.4 ± 16.7 (10–87)	50.3 ± 62.8 (9–342)	0.16 ^a^
Patients with a value >86 for men, and >30 U/L for women (%)	9 (23.1%)	11 (39.3%)	0.15 ^b^
**Albumin (g/L)**			
Mean ± SD (Actual range)	42.1 ± 3.2 (33–49)	42.3 ± 2.9 (36–48)	0.73 ^a^
Patients with an abnormal value (<39 or >50 g/L) (%)	5 (12.8%)	3 (10.7%)	0.79 ^b^
**Total Bilirubin (umol/L)**			
Mean ± SD (Actual range)	8.0 ± 3.9 (3.2–20.3)	7.4 ± 3.9 (4.0–18.6)	0.44 ^a^
Patients with a value >22.1 for men and >15.5 umol/L for women (%)	1 (2.6%)	1 (3.6%)	0.81
**hs-CRP (mg/L)**			
Mean ± SD (Actual range)	5.71 ± 5.57 (0.15–22.45)	7.57 ± 5.56 (0.4–20.05)	0.1 ^a^
Patients with a value 1–3 mg/L) (%)	21 (56.8%)	20 (71.4%)	0.22 ^b^
**HbA1c %**			
Mean ± SD (Actual range)	8.7 ± 2.1 (5.7–14.8)	8.3 ± 1.6 (5.7–12.1)	0.39 ^a^
Patients with a poorly controlled value (>7%) (%)	31 (79.5%)	21 (75.0%)	0.66 ^b^
**Fasting glucose mmol/L**			
Mean ± SD (Actual range)	10.4 ± 4.3 (6.0–22.6)	9.6 ± 4.1 (5.0–22.7)	0.39 ^a^
Patients with a poorly controlled value (≥7.2 mmlo/L) (%)	27 (69.2%)	20 (71.4%)	0.85 ^b^
**Insulin Umol/mL**			
Mean ± SD (Actual range)	13.1 ± 9.1 (2.7–42.8)	25.9 ± 27.4 (6.6–115.4)	**0.004** ^ a^

Glycated haemoglobin (HbA1c), HDL-Cholesterol (HDL-c), LDL-cholesterol (LDL-c), Aspartate aminotransferase (AST), Alanine aminotransferase (ALT), γ-glutamyl transferase (γ-GT), Alkaline Phosphatase (ALP), highly sensitive C-reactive protein (hs-CRP). Statistically significant values are shown in bold font. a *p*-value was obtained to compare the means of the two groups of people with T2DM with and without NAFLD using Independent T-test or Mann–Whitney U- when normality was not confirmed. b *p*-value was obtained to compare the proportions between the two groups using the Chi-Square test.

**Table 3:** Unadjusted and adjusted Odds Ratios (OR) with its 95% Confidence Interval (CI) for clinical and biochemical variables as predictors of NAFLD.

Covariate	Unadjusted	Adjusted for Age and WC
	OR (95% CI)	OR (95% CI)
** High DBP**		
Normal (reference)		
High DBP (≥90 mm\Hg)	11.93 (1.1, 135.8) ***p *= 0.049**	18.05 (1.32, 246.52) ***p* = 0.03**
**AST**		
AST (continuous)	1.095 (1.004, 1.1) ***p* = 0.041**	NS
** Low HDL-C**		
Normal (reference)		
Low HDL-c (<1.04 mmol/L for men and <1.3 mmol/L for women)	5.27 (1.58, 17.54) ***p* = 0.007**	9.92 (2.25, 43.61) ***p* = 0.002**

Variables are included based on the stepwise forward logistic regression model.

**Table 4:** Mean ± SD of fatty liver indices in T2DM patients with and without NAFLD, and the number of patients with high values of the indices.

Fatty Liver Indices	T2DM without NAFLD N = 39	T2DM with NAFLD N = 28	*p* Value
FLI			
Mean ± SD (Actual range)	82.5 ± 24.3 (9.1–99.8)	95.9 ± 6.6 (67.2–99.8)	**<0.001**
Patients with a high FLI (≥60) (%)	34 (87.2%)	28 (100%)	NS
Sensitivity	1		
Specificity	0.13		
HSI			
Mean ± SD (Actual range)	50.7 ± 9.9 (28.7–64.9)	56.7 ± 9.1 (37.7–70.1)	**0.022**
Patients with a high HSI (>36) (%)	35 (89.7%)	28 (100%)	NS
Sensitivity	1		
Specificity	0.103		
NAFLD-LFS			
Mean ± SD (Actual range)	0.56 ± 1.75 (−2.2–5.5)	2.92 ± 4.3 (−1.04–16.1)	**0.001**
Patients with a high NAFLD-LFS (>−0.64) (%)	32 (82.1%)	25 (96.2%)	NS
Sensitivity	0.962		
Specificity	0.154		
TyG index			
Mean ± SD (Actual range)	5.07 ± 0.35 (4.33–5.67)	5.11 ± 0.33 (4.61–5.79)	0.661
Patients with a high TyG index (>8.6) (%)	0 (0%)	0 (0%)	NS
Sensitivity	-		
Specificity	-		

Fatty liver index (FLI), hepatic steatosis index (HSI), non-alcoholic fatty liver disease-fatty liver score (NAFLD-FLS), triglyceride, and glucose (TyG).

**Table 5:** Area under the receiver operating characteristic (ROC) curve (AUC) and its 95% CI for different indices with NAFLD in patients with type 2 diabetes.

Indices	AUC	SE	95% CI	*p*-Value
FLI	0.774	0.060	0.657, 0.891	**<0.001**
HSI	0.640	0.071	0.501, 0.778	0.058
NAFLD-LFS	0.740	0.061	0.620, 0.86	**0.001**
TyG-Index	0.490	0.073	0.346, 0.633	0.888

FLI = Fatty liver index, HSI = Hepatic steatosis index, NAFLD-LFS = Nonalcoholic fatty liver disease-fatty liver score, TyG = Triglyceride, and glucose.

**Table 6:** The optimal cut-off values for liver fat indices and their sensitivity and specificity for the identification of NAFLD in diabetic patients.

Indices	Optimal Cut-Off Point	Sensitivity	Specificity	PLR	NLR
FLI	94.7	0.821	0.692	2.666	0.375
HSI	56	0.607	0.692	1.971	0.507
NAFLD-LFS	0.21	0.962	0.538	2.082	0.48

FLI = Fatty liver index, HSI = Hepatic steatosis index, NAFLD-LFS = Nonalcoholic fatty liver disease-fatty liver score, TyG = Triglyceride and glucose, PLR = Positive likelihood ratio, NLR = Negative likelihood ratio.

## Data Availability

The data used to support the findings of this study have been deposited in Food, Nutrition, and Lifestyle Unit, King Fahd Medical Research Centre, King Abdulaziz University Site. It can be accessed via the following link, http://www.kau.edu.sa/GetFile.aspx?id=313216&fn=NAFLD.rar accessed on 2 October 2022.
